# Rapid Evolution of the Mitochondrial Genome in Chalcidoid Wasps (Hymenoptera: Chalcidoidea) Driven by Parasitic Lifestyles

**DOI:** 10.1371/journal.pone.0026645

**Published:** 2011-11-02

**Authors:** Jin-Hua Xiao, Jing-Guo Jia, Robert W. Murphy, Da-Wei Huang

**Affiliations:** 1 Key Laboratory of Zoological Systematics and Evolution, Institute of Zoology, Chinese Academy of Sciences, Beijing, China; 2 Graduate School of the Chinese Academy of Sciences, Beijing, China; 3 College of Life Sciences, Hebei University, Baoding, China; 4 State Key Laboratory of Genetic Resources and Evolution, Kunming Institute of Zoology, Chinese Academy of Sciences, Kunming, China; 5 Department of Natural History, Royal Ontario Museum, Toronto, Ontario, Canada; Aarhus University, Denmark

## Abstract

Among the Chalcidoids, hymenopteran parasitic wasps that have diversified lifestyles, a partial mitochondrial genome has been reported only from *Nasonia*. This genome had many unusual features, especially a dramatic reorganization and a high rate of evolution. Comparisons based on more mitochondrial genomic data from the same superfamily were required to reveal weather these unusual features are peculiar to *Nasonia* or not. In the present study, we sequenced the nearly complete mitochondrial genomes from the species *Philotrypesis. pilosa* and *Philotrypesis* sp., both of which were associated with *Ficus hispida*. The acquired data included all of the protein-coding genes, rRNAs, and most of the tRNAs, and in *P. pilosa* the control region. High levels of nucleotide divergence separated the two species. A comparison of all available hymenopteran mitochondrial genomes (including a submitted partial genome from *Ceratosolen solmsi*) revealed that the Chalcidoids had dramatic mitochondrial gene rearrangments, involved not only the tRNAs, but also several protein-coding genes. The AT-rich control region was translocated and inverted in *Philotrypesis*. The mitochondrial genomes also exhibited rapid rates of evolution involving elevated nonsynonymous mutations.

## Introduction

In most animals, the mitochondrial genome is maternally inherited, generally nonrecombining with other mitochondrial lineages, and comprised of 13 protein-coding genes, 2 rRNAs and 22 tRNAs. The gene products work with nuclear-encoded mitochondrial proteins in the process of oxidative phosphorylation (OXPHOS) [1]. Due to its vital role in metabolism and relatively small size, the evolution of animal mitochondrial genomes remains intensively investigated. Complete mitochondrial genomes are known from many species of insects, yet few are recorded from the Hymenoptera. Although the mtDNA of the honeybee, *Apis mellifera*, has been available since 1993 [2], today few other complete hymenopteran genomes are known [3]. This situation may be due to two characteristics: the mitochondrial genome is extremely AT-rich rendering amplification and sequencing difficult and it has unusually high rates of substitution and frequent gene arrangements that confound primer design and amplification [4], [5]. For the Chalcidoidea, only a partial mitochondrial genome is known from *Nasonia*, and it has an unusually high accelerated rate of evolution and several unique gene rearrangements [6].

Mitochondrial genomes serve as good models for the study of molecular evolution and population genetics [7], [8], [9], [10]. Mitochondrial genome organization provides informative characters in sufficient quantity and quality for inferring phylogeny [1], [11], [12]. The high rate of evolution and genome reorganization of *Nasonia* may be typical of parasitic lifestyles in the Hymenoptera [5], [13], [14]. Parasitic chalcidoids have diversified lifestyles and mitochondrial genomic data from fig wasps that live inside the compact syconium of figs [15], might reveal features associated with their phyletic positions and lifestyles.

This study is concerned with three species of fig wasp: *Ceratosolen solmsi* and two species of *Philotrypesis* (*P. pilosa* and *Philotrypesis* sp.), all of which live in the same fig tree, *Ficus hispida*. Among these species, *C. solmsi* enjoys a mutualistic association with the fig; pollination occurs as it feeds on floret tissue. In contrast, species of *Philotrypesis* are parasitic on *C. solmsi*. Herein, sequences from the mitochondrial genome of *C. solmsi* (submitted) are compared to those of the parasites. The nearly complete mitochondrial genomes of the two species of *Philotrypesis* are highly diverged. Further, *Philotrypesis* also has unusual, dramatic gene rearrangments, not only in tRNAs, but also in several protein-coding genes. Below we discuss the relationship of accelerated mtDNA evolution and the unusual rearrangements with the evolution of fig wasps and the parasitic lifestyles in the Chalcidoidea.

## Materials and Methods

### Ethics statement

No experiments involving vertebrate samples were performed in this study. An ethics statement is not required for the experiments which only involve insects. The collections of wasps were permitted by the local park in Danzhou.

### Specimens and DNA extraction

Specimens of *P. pilosa* and *Philotrypesis* sp. were collected in 2008 from Danzhou, Hainan province, China. Wasps were identified and stored in 95% ethanol at −20°C. Images of the wasps used to confirm identification were captured by using a Nikon AZ100 microscope system. Only one individual from each species was chosen for DNA extraction by methods applicable for long PCR [16].

### Amplification and sequencing of mitochondrial genome fragments

We used degenerate primers modified from previous studies [17] to amplify the relatively conservative fragments *co1*–*nad3*, *nad5*–*cob*, and *nad1*–*12s*. Subsequently, species-specific primers were designed for the amplification of the regions between these fragments. Primer sequences were summarized in table S1. We used *HiFi Taq* (*TransGen*, Beijing, China) following the manufacturer's suggestions for PCR and the amplicons were either purified for direct sequencing or cloned for sequencing. The sequences were deposited in GenBank under the accession numbers JF808722 and JF808723.

### Genome annotation

The protein-coding and rRNA genes were identified by Blast searches in GenBank and aligned to the orthologous mitochondrial genes of *Nasonia* and *Apis mellifera*. Positional confirmation and annotation of the tRNAs was accomplished by using the online software of tRNAscan-SE 1.21 [18]. The detection of repeats used Tandem repeats finder [19].

### Genetic Divergence and Phylogeny

The combined sequences were aligned by using ClustalW and the software package DnaSP 5.0 [20] was used to compute nucleotide divergence, the ratio of Ka (the number of synonymous substation per synonymous site) and Ks (the number of nonsynonymous substation per nonsynonymous site). Based on genetic divergence, *Co1* and *co2* were inferred to be the most conserved protein-coding genes. They were employed for hypothesizing the phylogenetic relationships of the hymenoptera by using MrBayes 3.12 [21]. Twenty-four mitochondrial genome sequences (accession numbers for the downloaded genomes: EU746610-EU746612, NC_011923, NC_010967, NC_004529, NC_014295, NC_001566, NC_014272, NC_014278, NC_012708, NC_014677, NC_014669, NC_014672, NC_015075, NC_011520, NC_013238, NC_008323, NC_012688, NC_014485, NC_012689.) were used for phylogenetic inference and gene rearrangement analyses. *Orussus occidentalis* was chosen as the outgroup. We used The MrModeltest 2 to select the best-fit model for Bayesian analysis. Bayesian calculations used 1 million generations while sampling a tree every 100 generations. A 50% majority rule consensus tree was calculated from the sampled trees.

## Results

### Mitochondrial genomes of Philotrypesis

**Figure 1:**
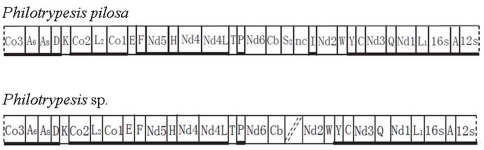
The mitochondrial genome of *Philotrypesis pilosa* and *Philotrypesis* sp. An unsequenced gap is located between *cob* and *nad2* in *Philotrypesis* sp.

For *P. pilosa*, 15,122 bp fragment of the mitochondrial genome was sequenced as follows: 13 protein-coding genes (*co3* incomplete), 16 tRNAs, 2 rRNAs (*12s* incomplete), and 1670 bp non-coding region between *trnS2* and *trnI*. The overall AT bias was 84.14%. For *Philotrpesis.* sp., we obtained two fragments of 8,567 bp and 3,330 bp. An unsequenced region occurred between *cob* and *nad2*. The sequenced fragments contained 13 protein-coding genes (*co3*, *cob*, and *nad2* incomplete), 14 tRNAs (missing *trnS2* and *trnI* compared to *P. pilosa*), and 2 rRNAs (*12s* incomplete) (Figure 1 and Table 1). The overall AT bias was 81.7%, slightly lower than in *P. pilosa*.

**Table 1:** Gene annotation and features for both genomes.

Gene	Strand	*Phipotrypesis pilosa*				*Philotrypesis* sp.			
		Length	Start	Stop	Space	Length	Start	Stop	Space
*co3*	-	incomplete	ATA		0	incomplete	ATA		12
*atp6*	-	675	ATG	TAA	-7	675	ATG	TAA	-7
*atp8*	-	165	ATT	TAA	1	165	ATT	TAA	0
*D*	-	65			9	70			6
*K*	+	69			4	70			4
*co2*	-	673	ATG	T-	69	675	ATT	TAA	68
*L2*	-	66			-4	66			92
*co1*	-	1545	ATG	TAA	6	1539	ATG	TAA	3
*E*	+	69			-1	68			0
*F*	-	66			0	65			0
*nad5*	-	1666	ATT	T-	0	1675	ATT	T-	0
*H*	-	67			1	67			3
*nad4*	-	1341	ATG	TAA	-7	1341	ATG	TAG	-7
*nad4l*	-	285	ATT	TAA	1	285	ATT	TAA	1
*T*	+	68			0	63			0
*P*	-	66			4	66			2
*nad6*	+	564	ATG	TAA	2	561	ATG	TAA	3
*cob*	+	1140	ATG	TAA	-2	incomplete	ATG		
*S2*	+	66			-1				
*nc*		1670			0				
*I*	-	67			62				
*nad2*	+	969	ATA	TAA	8	incomplete		T-	0
*W*	+	66			0	64			5
*Y*	-	66			67	65			65
*C*	-	65			1	64			0
*nad3*	-	334	ATT	T-	0	334	ATT	T-	0
*Q*	-	70			0	71			0
*nad1*	-	919	ATA	T-	0	919	ATA	T-	0
*L1*	-	63			0	64			0
*16s*	-	1309			0	1283			0
*A*	-	65			0	69			0
*12s*	-	incomplete				incomplete			

Table 1 compared the features of the mitochondrial genomes of both species. The two genomes had the same gene orientations, with 10 protein-coding genes and most of the tRNAs and both rRNAs located on the light strand. Only minor differences occurred. The lengths of most protein-coding gene were identical, except for *co1*, *nad5* and *nad6*. The size of 16s rRNA genes also differed. Only three tRNAs (*trnL2*, *trnH*, and *trnP*) of the 14 tRNAs had the same size. All predicted initiation codons translated to either methionine or isoleucine, as with most other bilateral animals [2]. *Co2* was the only gene to differ between the two species' start codons: *P. pilosa* had methionine (ATG) and *Philotrypesis* sp. isoleucine (ATT). Several genes had incomplete stop codons, a single T, which is common in animals. The products can be completed by posttranscriptional polyadenylation [6], [22]. As characteristic of mitochondrial genomes, inter-gene spaces were usually very short, yet several exceptions of up to 65–92 bp in length occurred in both genomes. The genome of *P. pilosa* had a 1670 bp non-coding region between *trnS2* and *trnI*. The corresponding region remained unknown in *Philotrypesis* sp.; we were unsure whether the latter species also had similar sequences or not. All of the predicted tRNAs had similar secondary structures (Figure S1).

### Accelerated rate of evolution

**Figure 2:**
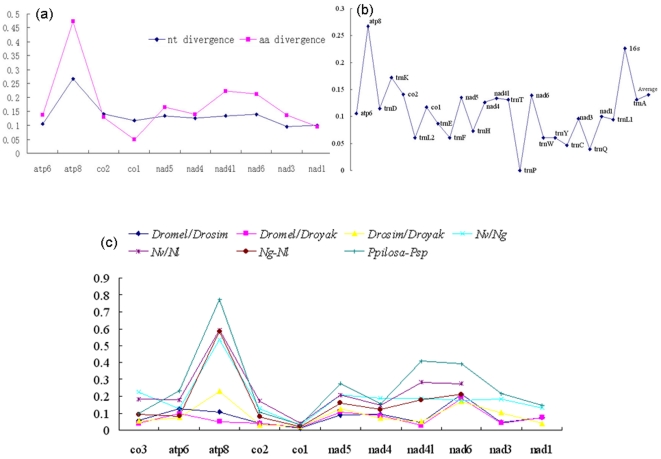
Genetic diversity of the two mitochondrial genomes of *Philotrypesis*. (a) Comparison of the protein-coding genes on both nucleotide and translated amino acid sequences; (b) Genetic divergence patterns throughout the genome. Pairwise sequence divergences are calculated with Bioedit and displayed as images suing Microsoft Excel. Genes on the x-axis are ordered according to their position in the genome of *Philotrypesis*. Numbers on the y-axis indicate the gene sequence divergence between the two species; 0.1  =  10% divergence or 90% similarity. (c) Ratio of Ka and Ks for 11 mitochondrial genes. Values of Ka and Ks are estimated with DnaSP v5 and corrected by the JC method.

The mitochondrial sequences diverge dramatically in the two species (Figure 2a and 2b). The average sequence divergence of the two genomes is 0.140. Protein-coding genes were more divergent than tRNAs; the highest divergence occurred in *atp8* (0.267). In contrast, *trnP* was identical in the two genomes. The large ribosomal RNA gene, *16s*, had the second largest divergence (0.226). This pattern indicated that the mitochondrial genomes of *Philotrypesis* either evolved rapidly or that the species diverged long ago. The former explanation seemed more likely because: 1) some of the genes were similar or even identical between the two species; 2) previous phylogenetic results based on the combination of mitochondrial (*cob*) and nuclear (*ITS2*) markers indicated that the two species may be sister taxa [23]; 3) morphological comparisons show that the two species were similar (Figure S2).

We compared the nucleotide and amino acid sequences of 10 protein-coding genes to further confirm that the mitochondrial genome of *Philotrypesis* was evolving rapidly (Figure 2a). Except for *co1*, *co2* and *nad1*, divergences in the amino acid sequences were greater than those of the nucleotide sequences. The greatest divergence occurred in *atp8*. The nucleotide divergence was 0.267, while the amino acid divergence was as high as 0.473. For genes showing greater amino acid sequence divergences, a greater number of nonsynonymous substitutions may have been accumulated, which would have accelerated their evolution by either positive or relaxed selection. In contrast, *co1* had a lower level of amino acid divergence (0.049) than nucleotide divergence (0.117), which indicated purifying selection. Further intra-specific comparisons may help determine whether the mitochondrial genomes have been evolving under neutral or positive selection.

A comparison of Ka/Ks ratios for 11 protein-coding genes was given for species in the genera *Drosophila*, *Nasonia* and *Philotrypesis* in Figure 2c and Table S2. *Nasonia* was reported to have dramatic higher Ka/Ks ratios than *Drosophila*, yet the two species of *Philotrypesis* had even higher ratios for most genes. This pattern indicated elevated evolutionary rates in the two species of *Philotrypesis*.

### Gene rearrangements

**Figure 3:**
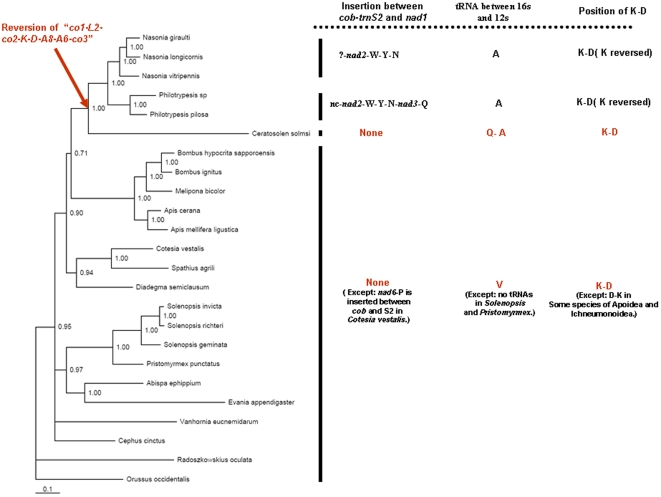
Bayesian estimation of phylogenetic relationships with mapped genome rearrangements for all hymenopterans having whole or partial mitochondrial genomic data. The major mitochondrial genome rearrangement events are compared and mapped out for *Ceratosolen*, *Nasonia* and *Philotrypesis*.

**Figure 4:**
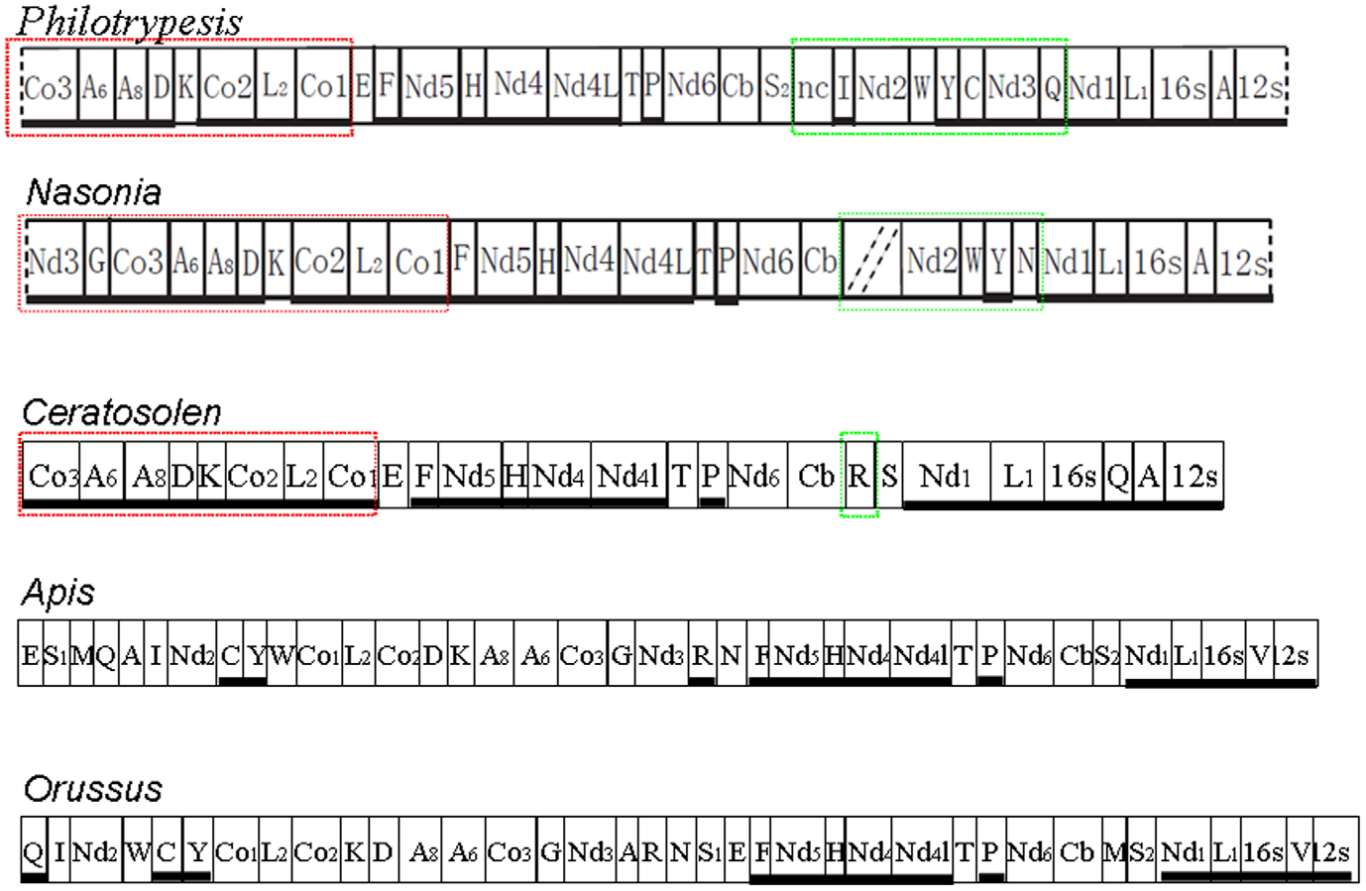
Mitochondrial genome organization in five genera. Red blocks indicate the large inversion specific to the Chalcidoidea, and the green blocks show the different and dramatically changed regions in the Chalcidoidea.

For a comparison of the gene orders, we made a NCBI search and downloaded all available hymenopteran mitochondrial genomes, and then constructed a phylogeny based on *co1* plus *co2* sequences. Orussus occidentalis was indicative of the ancestral state and used as the outgroup for all other hymenoptera mitochondrial genomes (Figure 3). The gene orders of five genomes are listed in Figure 4 (*Philotrypesis*, *Nasonia*, *Ceratosolen*, *Apis*, and *Orusses*), of which *Ceratosolen* was from the Agaonidae, and *Nasonia* and *Philotrypesis* represented the Pteramalidae. The later three genera were from the Chalcidoidea in the Proctotrupomorpha. Hymenopterans were reported to have many tRNA rearrangement events in their mitochondrial genomes [3]. However, the organizations of most of the species across the Apoidea, Ichneumnoidea, Vespoidea, Cephoidea, Evanioidea, and Proctotrupoidea were similar to that of the outgroup, Orussoidea, thus indicating little change. In comparison, chalcidoids had a series of mitochondrial gene rearrangements, the most striking being a large inversion in the region of at least five protein-coding genes. *Nasonia* had positional changes in at least seven protein-coding genes, and the large inversion affected six protein-coding genes [6]. The inversion affected only five genes in *Ceratosolen* and *Philotrypesis*.

Another dramatic change was located downstream of *cob*–*trnS2*. Compared to the Pteramalidae, *Ceratosolen* has a relatively ancient gene composition and order for *cob–trnR–trnS2–nad1*, with no insertion between *trnS2* and *nad1*. A large insertion occurred in members of the Pteramalidae. In *Nasonia*, at least *nad2*, *trnW*, *trnY*, and *trnN* were inserted before *nad1*. It was possible that additional genes are inserted in the region but that they did not amplify [6]. In *Philotrypesis*, a large insertion occurred between *trnS2* and *nad1*. The insertion was comprised of two protein-coding genes, five tRNAs and a non-coding region of 1670 bp.

Two additional apomorphic rearrangements occurred with tRNAs in the Pteramalidae relative to *Ceratosolen*. First, *trnK* was positioned in a ‘hot spot’ for rearrangements in the Hymenoptera [11]. Subsequent to the large inversion of *co1* and *co3* (or *nad3* in *Nasonia*), the orientation of *trnK* reversed in Pteramalidae but did not change in *Ceratosolen*. Second, among tRNAs occurring in the middle of the two rRNAs, the position of *trnV* appeared to be plesiomorphic because it occurred here in other arthropods [1]. As shown in Figure 3, the changes progressed from *trnV* in ancestral hymenoptera, to *trnQ*-*trnA* in *Ceratosolen*, and then to *trnA* in the Pteramalidae. When mapped onto the phylogeny of the hymenopterans (Figure 3), the apomorphic rearrangements clearly depicted the relationships of species in the Chalcidoidea.

### Non-coding sequences in Philotrypesis pilosa

**Figure 5:**
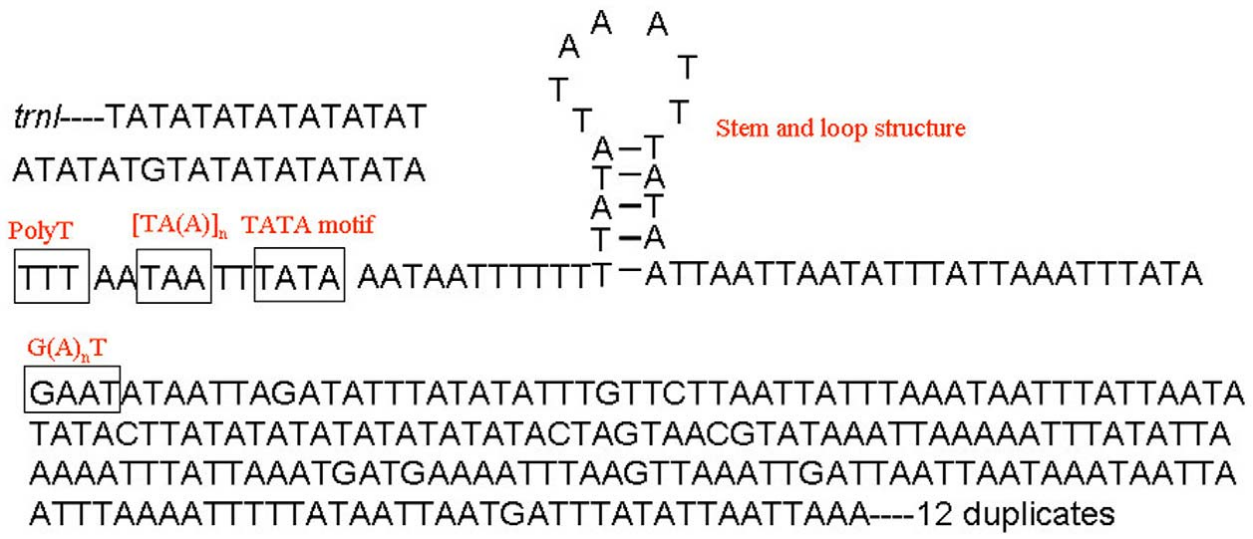
Structural elements of the AT-rich region in *Philotrypesis pilosa* shown as reverse and its complement. All the five elements characterized for the control region are indicated.

A 1670 bp fragment of non-coding sequences was resolved in the genome of *P. pilosa*. This fragment had an AT composition of 81.88%, a little less than the average AT bias in the entire genome. The fragment was located between *trnS2* and *trnI* and it was comprised of 12 duplicates plus a partial one for a total of 112 bp. Each of the 12 full duplicates had from one to five site mutations. The partial duplicate contained only 32 bp of the 5′ duplicate (Text S1). An AT-rich region followed the duplicates and it had an AT composition of 95.1%. This AT-rich region had five characteristic elements of the mitochondrial AT-rich control region believed to be involved in the regulation of transcription and control of DNA replication as follows: (1) a polyT stretch at the 5′end of the AT-rich region; (2) a [TA(A)]_n_-like stretch following the polyT stretch; (3) a stem-loop structure; (4) a TATA motif; and (5) a G(A)_n_T motif [24] (Figure 5). This control region was reversed and located on the light strand, a common characteristic of the Hymenoptera [25]. This inversion may have been associated with the inversion of large fragments of protein-coding genes, along with an inversion of the initiation of transcription sites.

## Discussion

In this study, we report the successful sequencing of the almost complete mitochondrial genomes from two species of *Philotrypesis* that shelter in the same figs. The major results are: (1) a high level of genetic divergence occurs between the two species; (2) like other mitochondrial genomes from the Chalcidoidea, *Philotrypesis* has dramatic gene rearrangments, not only in tRNAs, but also in several protein-coding genes; and (3) the AT-rich control region is translocated and inverted in *Philotrypesis*.

### Rapid genetic evolution in the Chalcidoidea and adaptation for endoparasitoids

Two species of *Philotrypesis* live sympatrically inside figs on the same tree, and they are phylogenetically and ecologically tightly associated with one another. They have an average mtDNA nucleotide divergence of 0.140. Most of the protein-coding genes have higher divergences in amino acid sequences than their corresponding nucleotide sequences. Both of these characteristics indicate that their mitochondrial genomes evolve rapidly. Oliveira et al. show that the mitochondrial genomes of closely related species of *Nasonia* are also very divergent and evolving very quickly [6]. Similarly, species of *Ceratosolen* have very divergent *co1* nucleotide sequences, and this is the most conserved gene in the group's mtDNA genome (unpublished data).

Insects in the Chalcidoidea have diverse parasitic lifestyles and perhaps their lifestyles are associated with the rapid rate of evolution of their mitochondrial genomes. This is suggested for parasitic hymenopterans [5], [13], [14]. Accelerated evolution of the mitochondrial genome may be associated with either the increased rate of speciation in parasitic Hymenoptera, adaptive radiations, or specific aspects of the endoparasitoid biology of the wasps [13].

### Dramatic gene rearrangements

Mitochondrial gene rearrangement, especially for tRNAs, is common in invertebrates [11]. However, changes in the relative positions of protein-coding genes are rare. *Nasonia* is the first species discovered to have a large inversion spanning at least six protein-coding genes [6].

The mitochondrial genomes of *C. solmsi* (submitted) and two species of *Philotrypesis* are now known. Similar to *Nasonia*, these species also have dramatic mitochondrial gene rearrangements and the extent of rearrangement is greatest in *Nasonia* and *Philotrypesis*. Members of the Pteramalidae have a translocation of the protein-coding gene *nad2*, the relative inversion of *trnK*. They also have a change of the tRNA between two rRNAs. These rearrangements are consistent with the observation that mitochondrial gene rearrangments can be used in phylogenetic reconstructions, just like genome ‘morphology’ [12], because *C. solmsi* is in the Agaonidae and *Nasonia* and *Philotrypesis* are in the Pteramalidae (Figures 3 and 4).

A comparison of gene order among *Orussus*, *Apis*, *Ceratosolen*, and the Pteramalidae reveals that *Ceratosolen* has more plesiomorphic character states than species in the Pteramalidae (Figure 4). The most striking gene reorganization occurs downstream of *cob*, where *Ceratosolen* has an insertion of only *trnR* while the Pteramalidae has a large inserted fragment comprising several tRNAs plus one or two protein-coding genes. Further, whereas the orientation of *trnK* is not changed in *Ceratosolen*, it is reversed in the Pteramalidae and subsequent to the large inversion event of *co1* to *co3* (or to *nad3* in *Nasonia*) in the Chalcidoidea. A third shift involves the tRNAs between the two rRNAs. The *trnV* in the outgroup taxa *Orussus* and *Apis* shifts to *trnQ*-*trnA* in *Ceratosolen*, and then to *trnA* in the Pteramalidae. Phylogenetic studies indicate that fig wasps do not share a common ancestor: Different lineages of chalcids are involved in many independent colonization events and the family Agaonidae may be older than all other families of fig wasps [26], [27]. Our data on mitochondrial gene rearrangement supports an older age for the Agaonidae relative to *Philotrypesis*. The latter appears to have colonized figs after the origin of the fig-wasp association (Figure 3).

The pattern of accelerated gene rearrangement may be correlated with parasitic lifestyles, though this is still debated [3], [28]. The rate of gene rearrangements is correlated with mitochondrial genetic diversity [29], [30] and our data show that chalcids have a rapid rate of genetic evolution. We emphasize that among the three genera we examined from the Chalcidoidea, *Ceratosolen* has the least amount of rearrangements, and *Nasonia* has fewer rearrangements than *Philotrypesis*. For example, *nad3* is not translocated in *Nasonia* but it changes in *Philotrypesis*. With respect to lifestyles, *Ceratosolen* is a galler that feeds on the floret tissues of the fig and acts as one partner in the mutualism system of fig and wasp. In contrast, *Nasonia* and *Philotrypesis* are both endoparasites that feed on other insects. Indeed, *Philotrypesis* is parasitic to *Ceratosolen* in the floret inside the syconium, a compact and dark world, and it lives in a distinctly different oxygen environment from *Nasonia*.

In conclusion, our study presents two new mitochondrial genomes from chalcidoids including the two species of *Philotrypesis*. It evaluates these genomes with respect to those of other insects in the Chalcidoidea. This comparison leads to the discovery of rapid rates of evolution involving elevated nonsynonymous mutations and unusual, dramatic gene rearrangements. These changes may be correlated with parasitic lifestyles including the evolution of fig wasps in the peculiar syconia environment.

## Supporting Information

Table S1 **Primers used in the amplification of the mitochondrial genomes.**
(DOC)

Table S2 **Sequence divergence estimated by Ka/Ks for 11 mitochondrial genes.**
(DOC)

Figure S1 **Predicted secondary structure of the tRNAs in both mitochondrial genomes.**
(DOC)

Figure S2 **The morphological comparisons of the two** ***Philotrypesis*** **species.** a-c: *Philotrypesis pilosa*; d-f: *Philotrypesis* sp. (a,d: body of the female; b,e: body of the male; c,f: dorsum of male's head). The two species are very similar except some minor differences As follows: female *Philotrypesis pilosa*, ratio of seventh and eighth Gastral tergum length about 3, and ovipositor length twice body length; female *Philotrypesis* sp., ratio of the seventh and eighth Gastral tergum length about 6, and ovipositor length 3 times body length; male *Philotrypesis pilosa*, malar space obviously shorter than length of eyes; male *Philotrypesis* sp., malar space larger or approximately equal to length of eyes.
(TIF)

Text S1 **The 1670 bp non-coding region between tRNA-S2 and tRNA-I in** ***Philotrypesis pilosa*** **.**
(DOC)
