# Supplementary material for: Rapid Evolution of the Mitochondrial Genome in Chalcidoid Wasps (Hymenoptera: Chalcidoidea) Driven by Parasitic Lifestyles
Source: PLoS One. 2011 Nov 2;6(11):e26645. doi: 10.1371/journal.pone.0026645 (PMC3206819; doi:10.1371/journal.pone.0026645)
Supplement: Table S1 — Primers used in the amplification of the mitochondrial genomes. (DOC) [file pone.0026645.s001.doc]

| Primer pairs | Spanning region | Species | Sequences |
| --- | --- | --- | --- |
| PpCo1/PpCo3 | ***co1*-*co3*** | *Pp* | 5’-TTCAAGAATATTTGT**G**GGAACTGG-3’  5’-AAAATAGA**A**CCATA**A**AC**A**GAATC**T**GC-3’ |
| PspCo1/PspNCo3 |  | *P*sp | 5’-TTCAAGAATATTTGT**T**GGAACTGG-3’  5’-AAAATAGA**T**CCATA**T**AC**T**GAATC**A**GC-3’ |
| pco1F/pn5R | ***co1*-*nad5*** | *Pp*&*P*sp | 5’-ATAAATCAACWGAWGGMCC-3’  5’-TGATAAAGGTTGAAGWGAA-3’ |
| PpNCo1/PpCytB | ***co1*-*cob*** | *Pp* | 5’-**A**GATAA**A**CC**T**ATAAT**T**CCTGCTC-3’  5’-**T**GATATTTG**G**CCTCAAGGTAGAAC-3’ |
| PspNCo1/PspCytB |  | *P*sp | 5’-**T**GATAA**C**CC**—A**TAAT**C**CCTGCTC-3’  5’-**A**GATATTTG**C**CCTCAAGGTAGAAC-3’ |
| PpcytbF/PpcytbR | ***cob*-*cob*** | *Pp* | 5’-ATTCAAGATGTAAATTATGGAT-3’  5’-TATAAATTGCAAATAGATGC-3’ |
| PpcoB/Ppn2 | ***cob*-*nad2*** | *Pp* | 5’-ATGTCCAGTTGAAATACCGT-3’  5’-AGGGGGTAATGATGCTAAAG-3’ |
| pn2F/Ppn1 | ***nad2*-*nad1*** | *Pp* | 5’-TTCWTTAGCWTCAWTHCCHCC-3’  5’-TATTACAACCTTTTAGTGATGC-3’ |
| pn2F/PSPn1R |  | *P*sp | 5’-TTCWTTAGCWTCAWTHCCHCC-3’  5’-AGTTGGTTTTATTGGATTATTACA-3’ |
| PpNd1F/Pp12sR | ***nad1*-*12s*** | *Pp*&*P*sp | 5’-ATTTAATTGCATCACTAAAAGGTTGT-3’  5’-AATGAGTAAGTCGTAACAAAGTAA-3’ |

Notes: Primers used in the amplification of the mitochondrial genomes.

The different sites between two species primers are indicated, with the read paned sites specific in *Philotrypesis pilosa* (*Pp*), while the blue sites with underlines specific in *Philotrypesis* sp.(*P*sp).
