# Supplementary material for: Rapid Evolution of the Mitochondrial Genome in Chalcidoid Wasps (Hymenoptera: Chalcidoidea) Driven by Parasitic Lifestyles
Source: PLoS One. 2011 Nov 2;6(11):e26645. doi: 10.1371/journal.pone.0026645 (PMC3206819; doi:10.1371/journal.pone.0026645)
Supplement: Table S2 — Sequence divergence estimated by Ka/Ks for 11 mitochondrial genes. (DOC) [file pone.0026645.s002.doc]

|  | *co3* | *atp6* | *atp8* | *co2* | *co1* | *nad5* | *nad4* | *nad4l* | *nad6* | *nad3* | nad1 |
| --- | --- | --- | --- | --- | --- | --- | --- | --- | --- | --- | --- |
| *Dromel*/*Drosim* | 0.059 | 0.1247 | 0.109 | 0.0426 | 0.0123 | 0.0906 | 0.0925 | 0.0465 | 0.2101 | 0.0453 | 0.0748 |
| *Dromel*/*Droyak* | 0.0408 | 0.0968 | 0.0512 | 0.038 | 0.0169 | 0.1075 | 0.0848 | 0.0297 | 0.1912 | 0.0445 | 0.0761 |
| *Drosim*/*Droyak* | 0.0548 | 0.075 | 0.2309 | 0.0322 | 0.0202 | 0.1256 | 0.07 | 0.0489 | 0.1673 | 0.1034 | 0.04 |
| *Nv*/*Ng* | 0.2258 | 0.1283 | 0.5332 | 0.1283 | 0.0271 | 0.2082 | 0.1909 | 0.1873 | 0.177 | 0.1821 | 0.1321 |
| *Nv*/*Nl* | 0.1843 | 0.1799 | 0.5916 | 0.1714 | 0.0383 | 0.2073 | 0.1485 | 0.2843 | 0.2776 | ＊ | ＊ |
| *Ng*-*Nl* | 0.0932 | 0.0819 | 0.5846 | 0.0803 | 0.0214 | 0.1605 | 0.1211 | 0.1807 | 0.2111 | ＊ | ＊ |
| *Ppilosa*-*P*sp | 0.0956 | 0.2341 | 0.7723 | 0.1081 | 0.0323 | 0.2749 | 0.1537 | 0.4098 | 0.3918 | 0.2148 | 0.1467 |

Notes: Comparisons are made between species from the same genus for *Drosophila*, *Nasonia*, and *Philotrypesis* respectively. Genes are ordered according to their position in the mitochondrial genome of *Philotrypesis*. Values of Ka/Ks are estimated with DnaSP v5 and corrected by the JC method. (Abbreviation: *Dromel*: *Drosophila melanogaster*, *Drosim*: *Drosophila simulans*, *Droyak*: *Drosophila yakuba*, *Nv*: *Nasonia vitripennis*, *Ng*: *Nasonia giraulti*, *Nl*: *Nasonia longicornis*, *Ppilosa*: *Philotrypesis pilosa*, *P*sp: *Philotrypesis* sp..)

＊We do not make the comparisons because the sequences are very short for *Nasonia longicornis*.
