# Supplementary material for: Rapid Evolution of the Mitochondrial Genome in Chalcidoid Wasps (Hymenoptera: Chalcidoidea) Driven by Parasitic Lifestyles
Source: PLoS One. 2011 Nov 2;6(11):e26645. doi: 10.1371/journal.pone.0026645 (PMC3206819; doi:10.1371/journal.pone.0026645)
Supplement: Figure S1 — Predicted secondary structure of the tRNAs in both mitochondrial genomes. (DOC) [file pone.0026645.s003.doc]

b: Predicted secondary structure of the tRNAs in *Philotrypesis.* Sp.


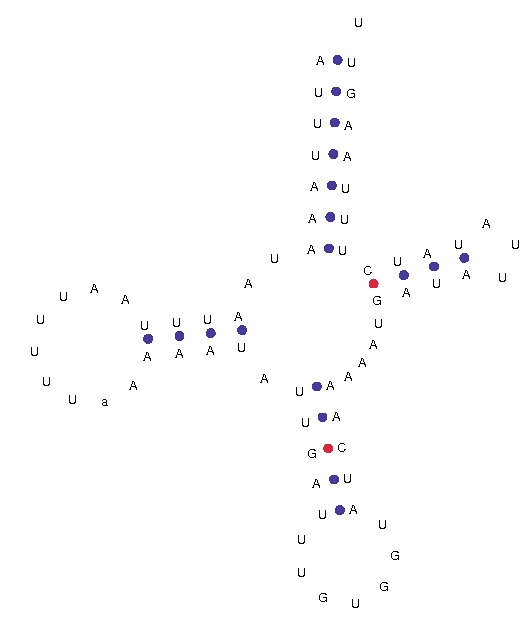


His(H)-GTG


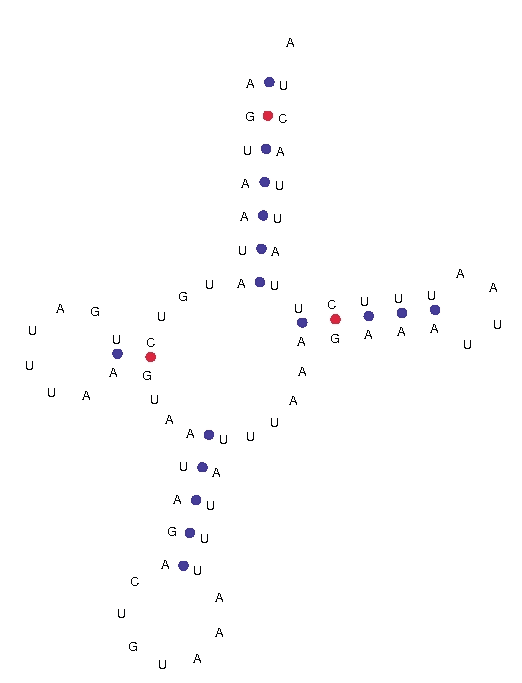


Tyr(Y)-GTA


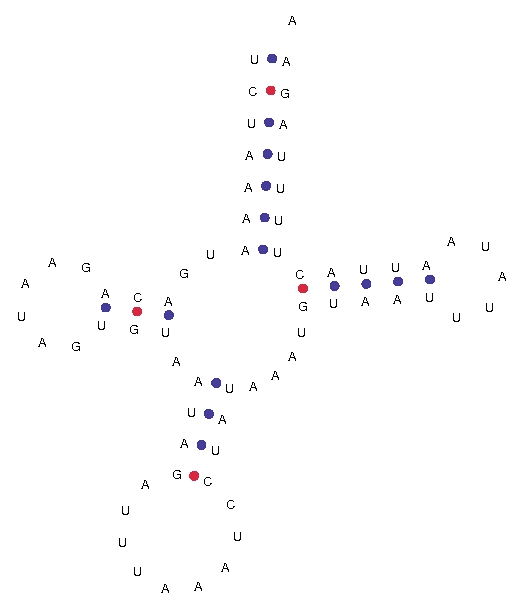


Leu(L2)-TAA


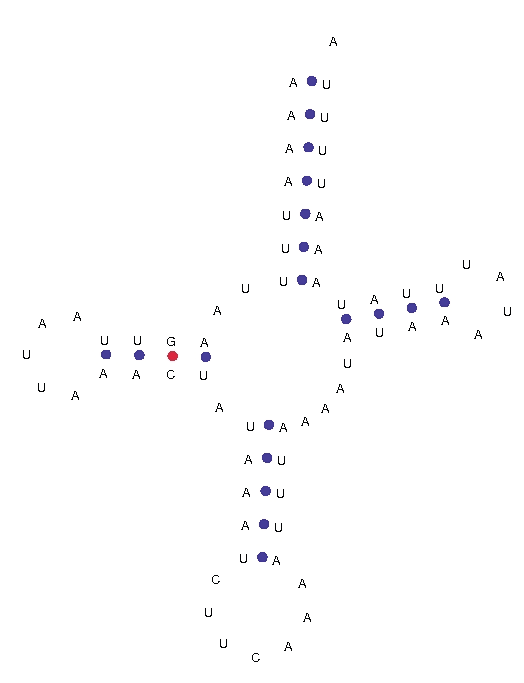


Trp(W)-TCA


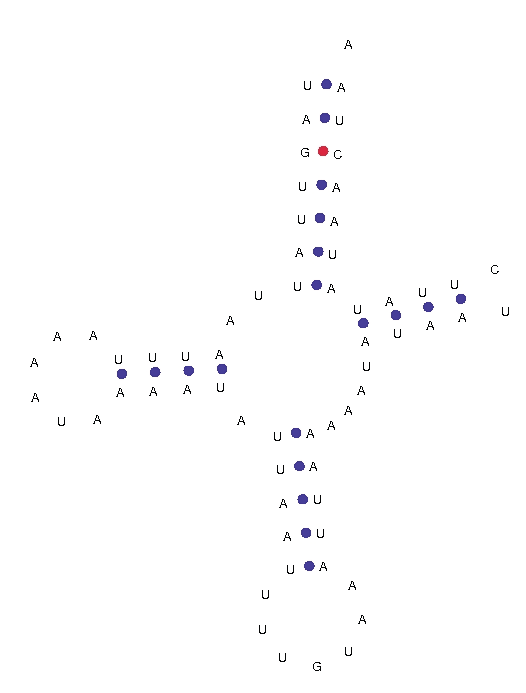


Thr(T)-TGT


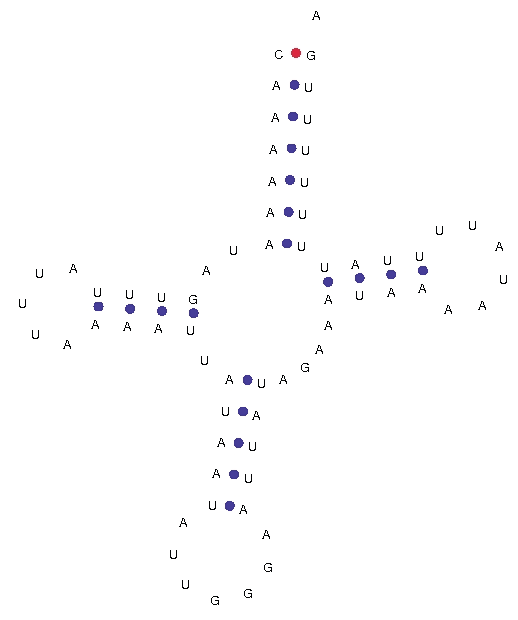


Pro(P)-TGG


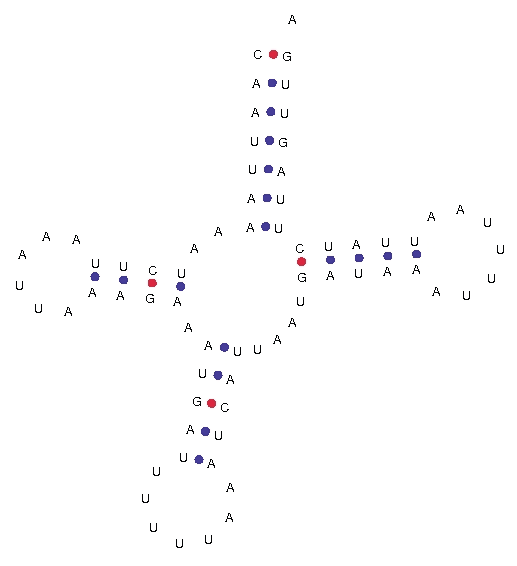


Lys(K)-TTT


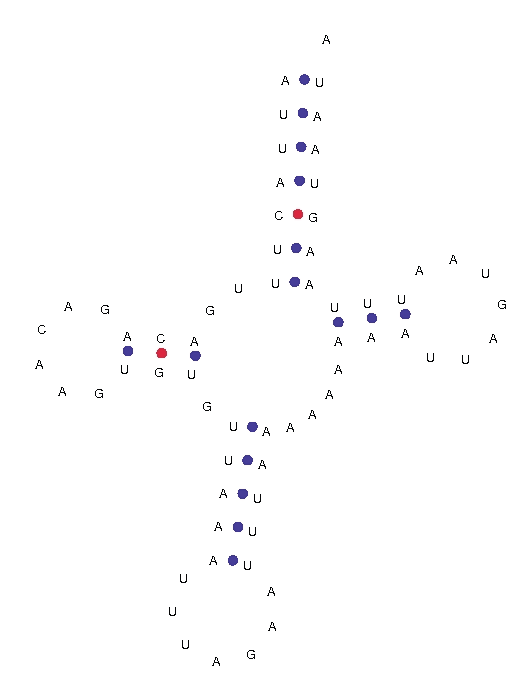


Leu(L1)-TAG


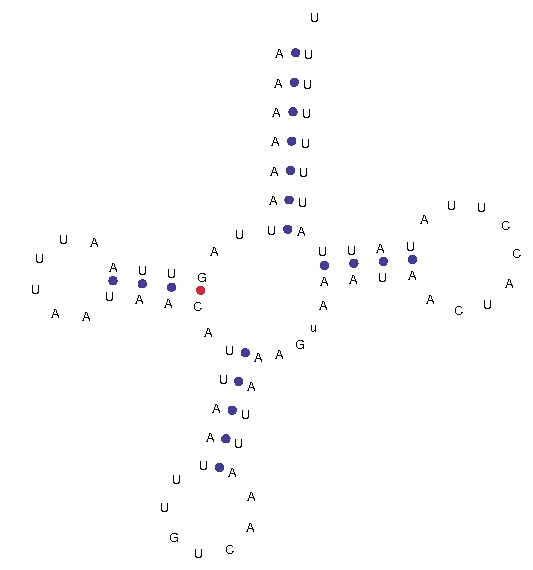


Asp(D)-GTC


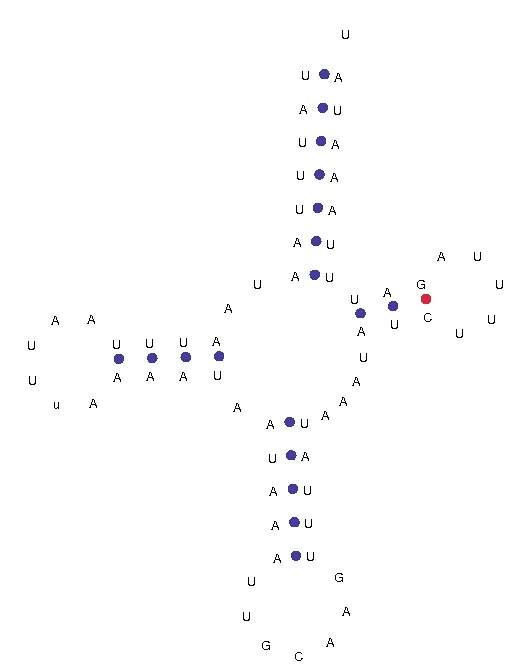


Cys(C)-GCA


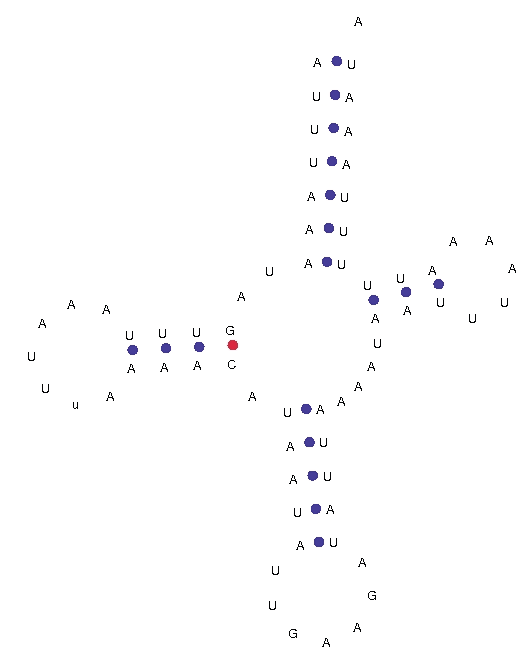


Phe(F)-GAA


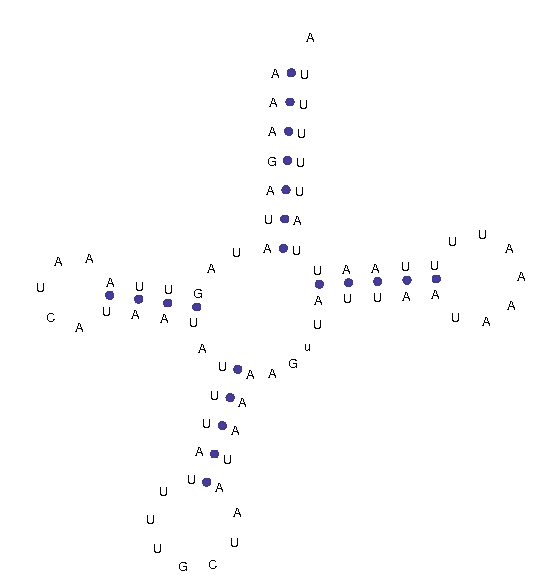


Ala(A)-TGC


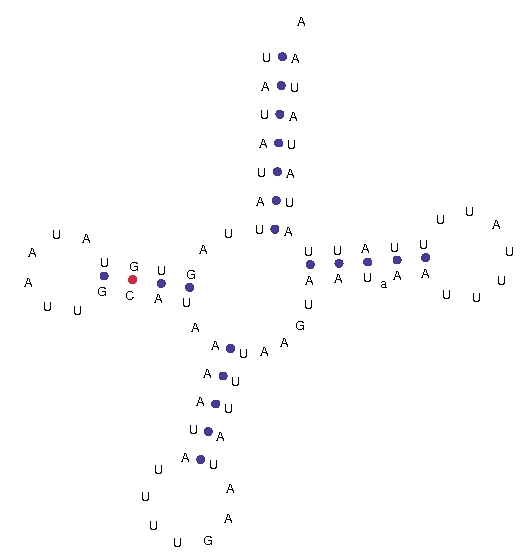


Gln(Q)-TTG


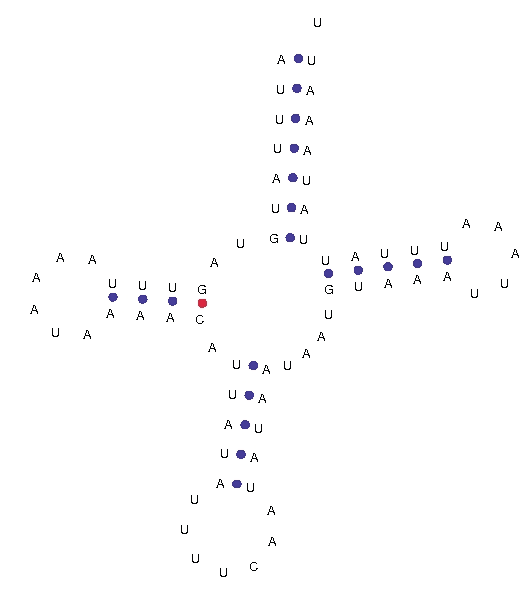


Glu(E)-TTC

a: Predicted secondary structure of the tRNAs in *P. pilosa*


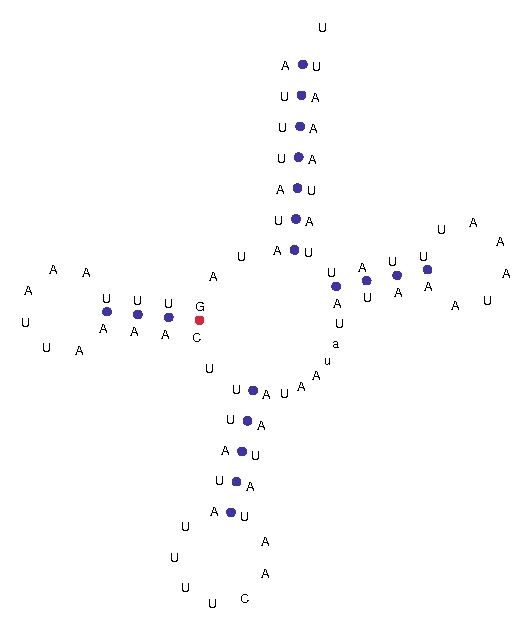


Glu(E)-TTC


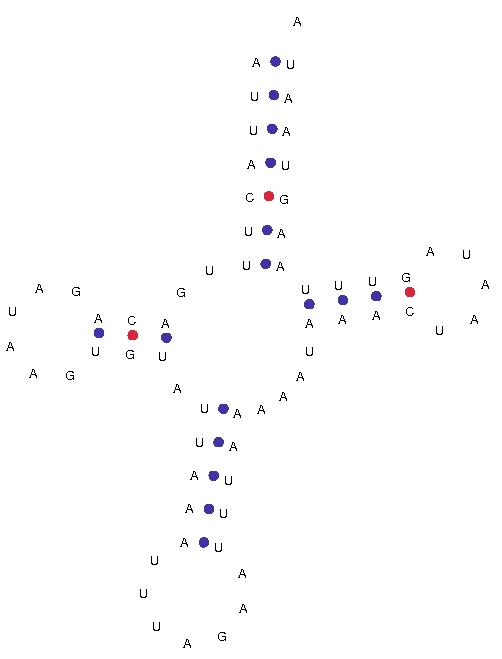


Leu(L1)-TAG


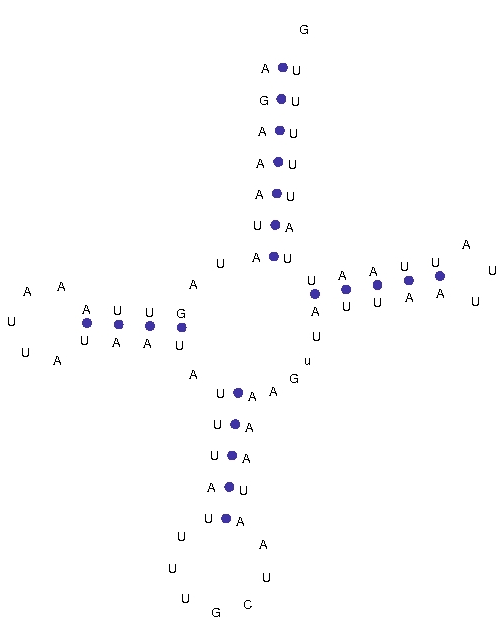


Ala(A)-TGC


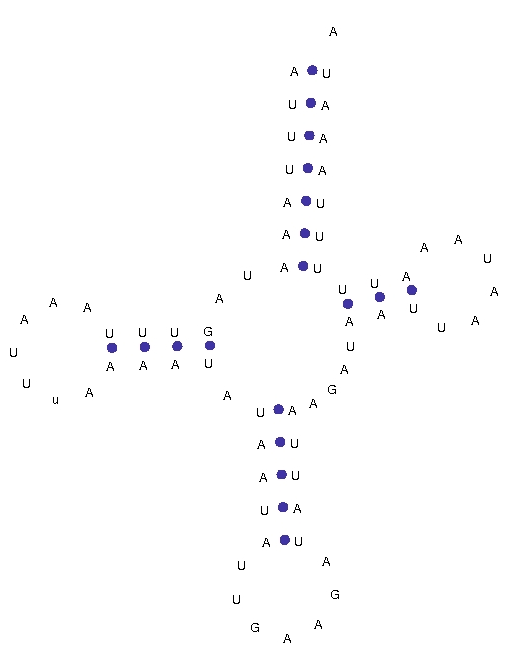


Phe(F)_GAA


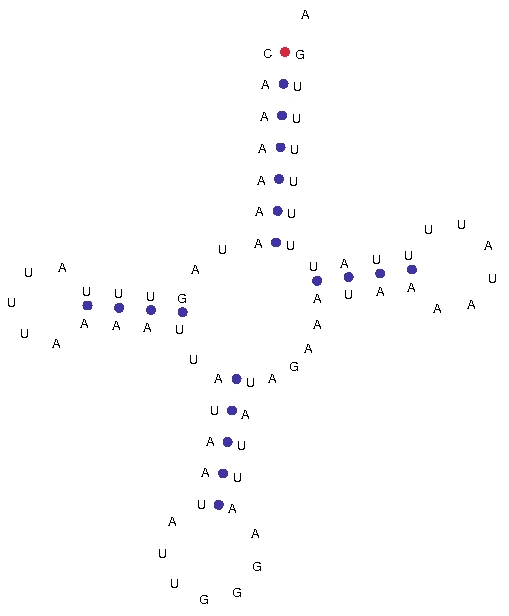


Pro(P)_TGG


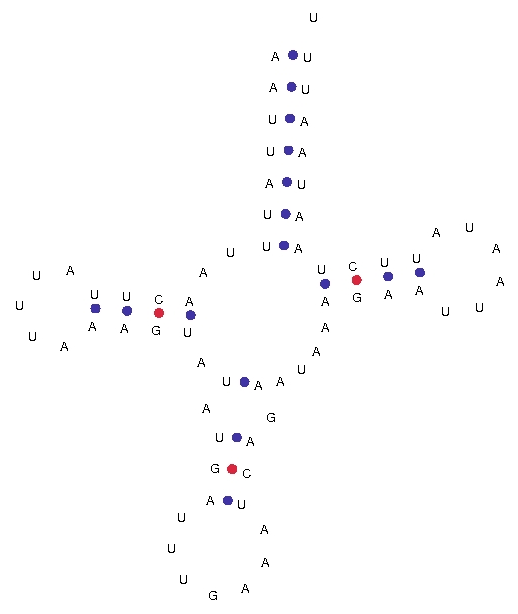


Ser(S2)-TGA


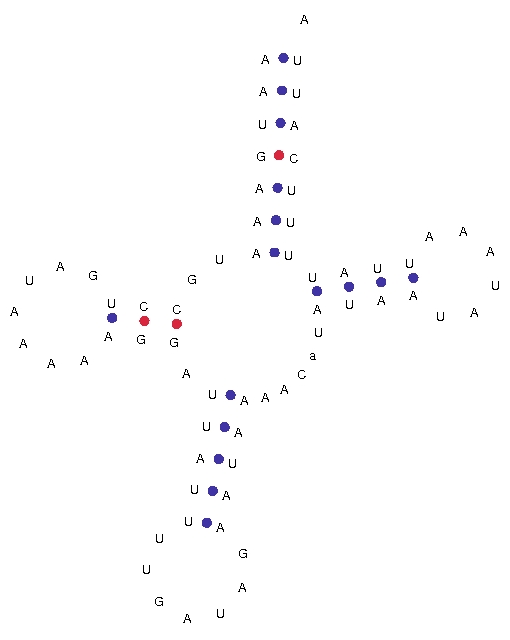


Ile(I)-GAT


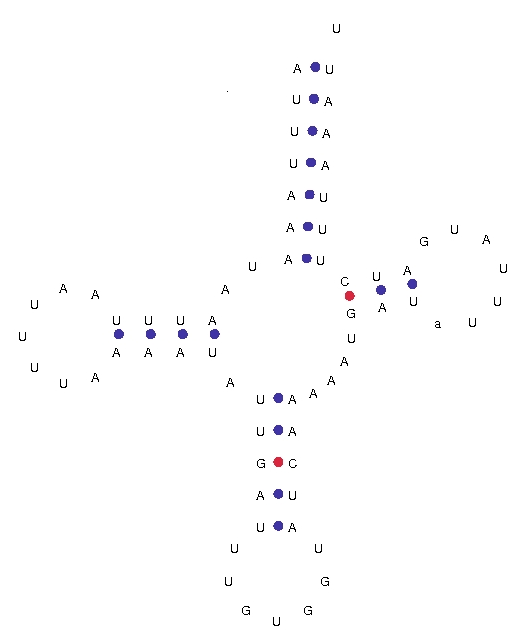


His(H)-GTG


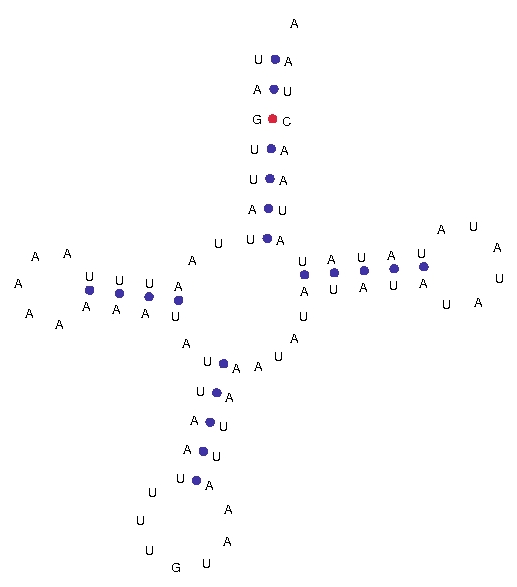


Thr(T)-TGT


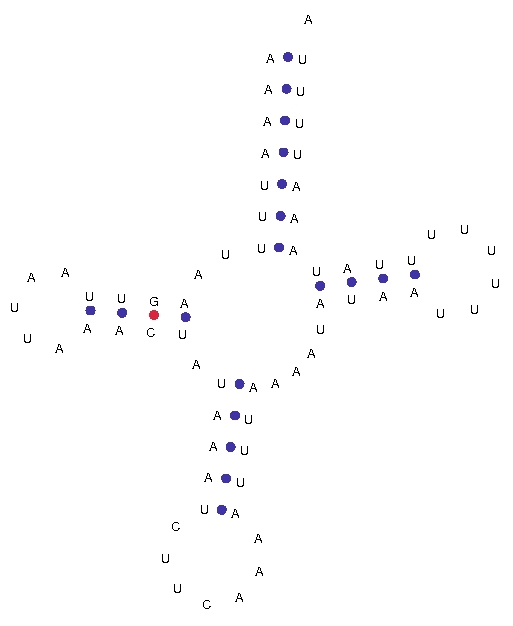


Trp(W)-TCA


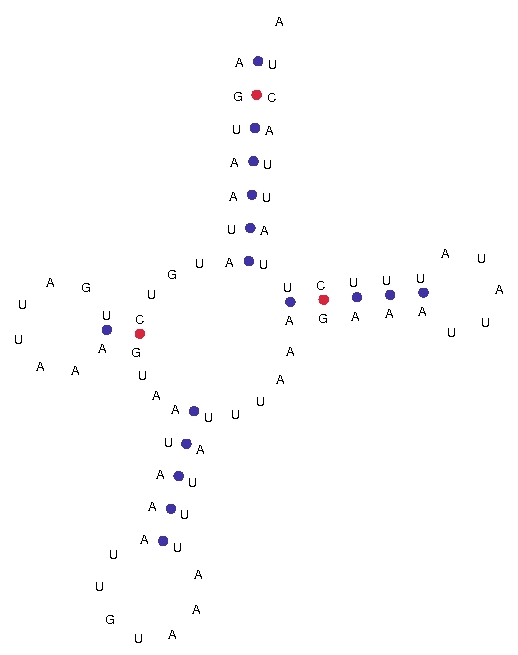


Tyr(Y)-GTA


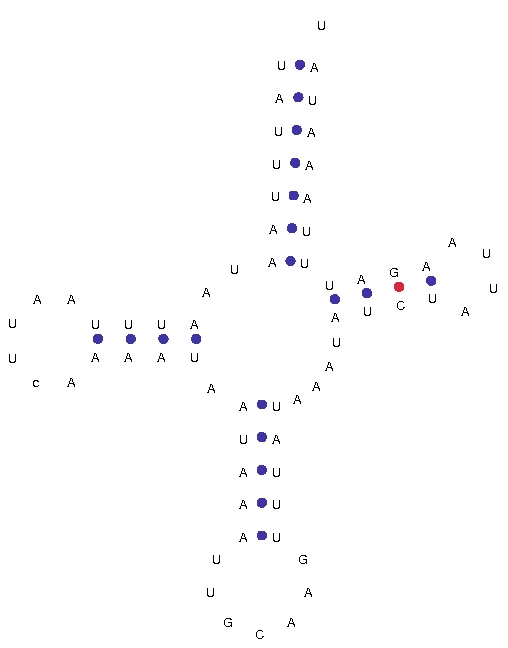


Cys(C)-GCA


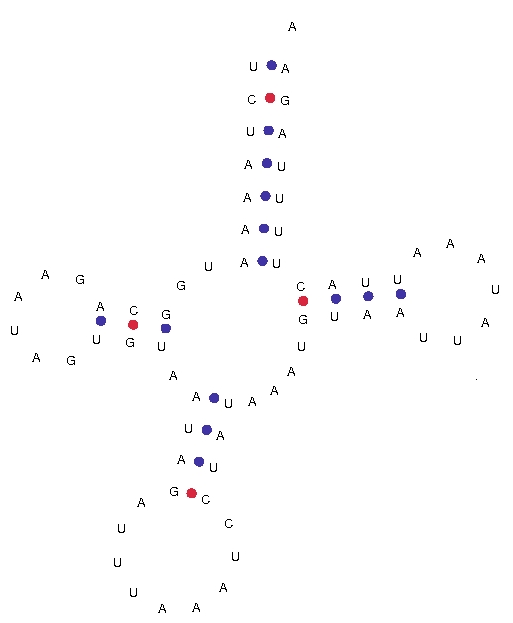


Leu(L2)-TAA


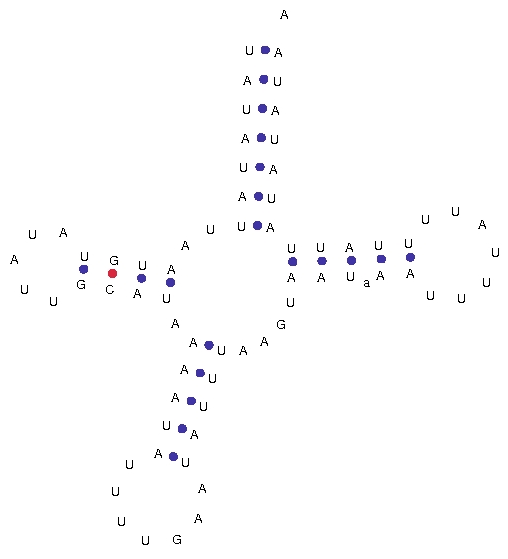


Gin(Q)-TTG


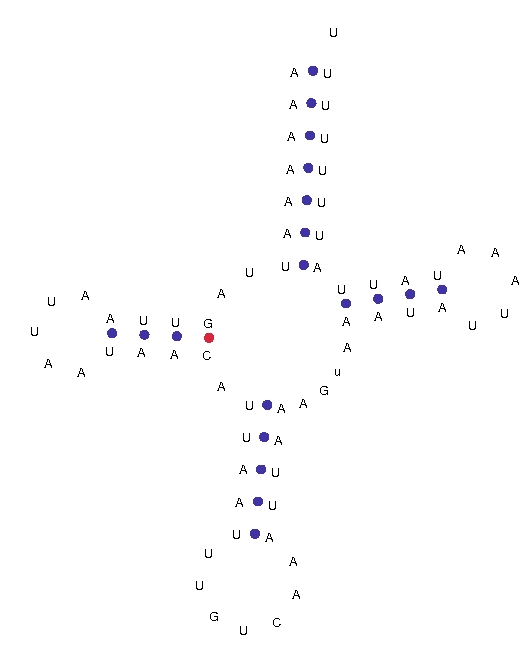


Asp(D)-GTC


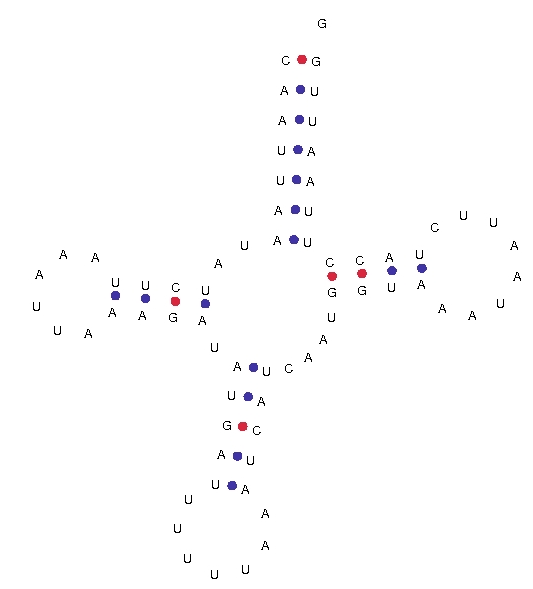


Lys(K)-TTT
