# Supplementary figures and images for: Rapid Evolution of the Mitochondrial Genome in Chalcidoid Wasps (Hymenoptera: Chalcidoidea) Driven by Parasitic Lifestyles
Source: PLoS One. 2011 Nov 2;6(11):e26645. doi: 10.1371/journal.pone.0026645 (PMC3206819; doi:10.1371/journal.pone.0026645)

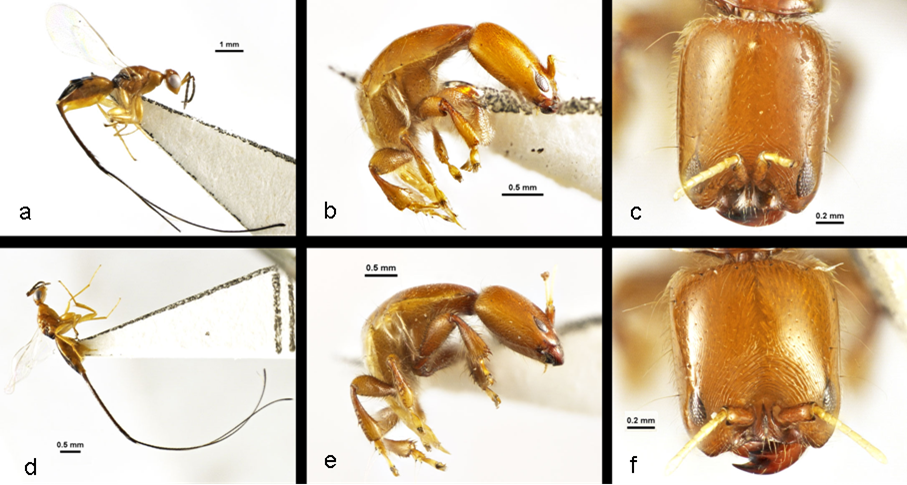

Supplement: Figure S2 — The morphological comparisons of the two Philotrypesis species. a-c: Philotrypesis pilosa; d-f: Philotrypesis sp. (a,d: body of the female; b,e: body of the male; c,f: dorsum of male's head). The two species are very similar except some minor differences As follows: female Philotrypesis pilosa, ratio of seventh and eighth Gastral tergum length about 3, and ovipositor length twice body length; female Philotrypesis sp., ratio of the seventh and eighth Gastral tergum length about 6, and ovipositor length 3 times body length; male Philotrypesis pilosa, malar space obviously shorter than length of eyes; male Philotrypesis sp., malar space larger or approximately equal to length of eyes. (TIF) [file pone.0026645.s004.tif]
