# Supplementary material for: Rapid Evolution of the Mitochondrial Genome in Chalcidoid Wasps (Hymenoptera: Chalcidoidea) Driven by Parasitic Lifestyles
Source: PLoS One. 2011 Nov 2;6(11):e26645. doi: 10.1371/journal.pone.0026645 (PMC3206819; doi:10.1371/journal.pone.0026645)
Supplement: Text S1 — The 1670 bp non-coding region between tRNA-S2 and tRNA-I in Philotrypesis pilosa . (DOC) [file pone.0026645.s005.doc]

CTAATAATTTTAATAATTCACTAAATTTAACACCTCAGTTAAATTTTACTATATACCATTTAACTTATTTCTCCCCACAAAAATAAGTACTACAATATACAATAGGTTTATA

CTAATAATATTAATAATTCACTAAATTTAACACCCCTGTAAAATTTTACTATATACCATTTAACTTATTTCTCCCCACAAAAATAAGTACTACAATACACAATAGGTTTATA

TTAATAATATTAATAATTCACTAAATTTAACACCTCAGTTAAATTTTACTATATACCATTTAACTTATTTCTCCCCACAAAAATAAGTACTACGATATACAATAGGTTTATA

CTAATAATATTAATAATTCACTAAATTTAACACCTCAGTAAAATTTTACTATATACCATTTAACTTATTTCTCCCCACAAAAATAAGTACTACAATATACAATAGGTTTATA

CTAATAGTATTAATAATTCAATAAATTTAATA_CTCAGTTAAATTTTACTATATACCATTTAACTTATTTCTCCCCACAAAAATAAGTACTACAATATACAATAGGTTTATG

CAAATAATATTAATAATTCACTAAATTTAACACCTCAGTTAAATTTTACTATATACCATTTAACTTATTTCTCCCCACAAAAATAAGTACTACAATATACAATAGGTTTATA

CTAATAATATTAATAATTCACTAAATTTAACACCTCAGTTAAATTTTACTATATACCATTTAACTTATTTCTCCCCACAAAAATAAGTACTACAATACACAATAGGTTTATA

CTAATAATATTAATAATTCATTAAATTTAACACGTCAGTTAAATTTTACTATATACCATTTAACTTATTTCTCCCCACAAAAATAAGTACTACAATATACAATAGGTTTATA

CTAATAATATTAATAATTCACTAAATTTAACACCTCAGTTAAATTTTACTATATACCATTTAACTTATTTCTCCCCACAAAAATAAGTACTACAATATACAATAGGTTTATA

CTAATAATATTAATAATTCAATAAATTTAATA_CTCAGTTAAATTTTACTATATACCATTTAA_TTAATTTCTCCCCACAAAAATAAGTACTACAATATACAATAGGTTTATA

CTAATAATATTAATAATTCACTAAATTTAACACCTCAGTTAAATTTTACTATATACCATTTAACTTATTTCTCCCCACAAAACTAAGTACTACAATATACAATAGGTATATA

ATAATAATATTAATAATTCACTAAATTTAACACCTCAGTTAAATTTTACTATATACCATTTAACTTATTTCTCCCCACAAAAATAAGTACTACAATATACAATAGGTTTATA

CTAATAATATTAATAATTCACTAAATTTAACA

TTTAATTAATATAAATCATTAATTATAAAAATTTTAAATTAATTATTTATTAATTAATCAATTTAACTTAAATTTTCATCATTTAATAAATTTTTAATATAAATTTTTAATTTATACGTTACTAGTATATATATATATATATAAGTATATATTAATAAATTATTTAAATAATTAAGAACAAATATATAAATATCTAATTATATTCTATAAATTTAATAAATATTAATTAATTATAAATTTAATATAAAAAAAATTATTTATAAATTATTAAATATATATATATACATATATATATATATATATATA

Notes: The 1670 bp non-coding region between tRNA-S2 and tRNA-I in *Philotrypesis pilosa.* The underlined region is the 112 bp duplicate element. There are altogether 12 full duplicates of this element, with only several mutation sites among them (The red characters in panes). After the 12 duplicates, there exists a 32 bp fragment of the 5’ of the duplicate. The fragment with yellow background is the reversed control region as depicted in Figure 4.
